# Bolstering the secretion and bioactivities of umbilical cord MSC-derived extracellular vesicles with 3D culture and priming in chemically defined media

**DOI:** 10.1186/s40580-022-00349-z

**Published:** 2022-12-19

**Authors:** Jun Yong Kim, Won-Kyu Rhim, Seung-Gyu Cha, Jiwon Woo, Joo Youn Lee, Chun Gwon Park, Dong Keun Han

**Affiliations:** 1 Department of Biomedical Science, CHA University, 335 Pangyo-ro, Bundang-gu, Seongnam-si, Gyeonggi-do 13488 Republic of Korea; 2 Department of Biomedical Engineering, Sungkyunkwan University (SKKU), 2066 Seobu-ro, Jangan-gu, Suwon-si, Gyeonggi-do 16419 Republic of Korea; 3 Intelligent Precision of Healthcare Convergence, SKKU Institute for Convergence, Sungkyunkwan University (SKKU), 2066 Seobu-ro, Jangan-gu, Suwon-si, Gyeonggi-do 16419 Republic of Korea; 4 Xcell Therapeutics, 333, Yeongdong-daero, Gangnam-gu, Seoul, 06188 Republic of Korea

**Keywords:** Extracellular vesicle, Nano vesicles, Mesenchymal stem cell, Chemically defined media, 3D spheroids, Mesenchymal stem cell priming, Regenerative activity

## Abstract

Human mesenchymal stem cells (hMSCs)-derived extracellular vesicles (EVs) have been known to possess the features of the origin cell with nano size and have shown therapeutic potentials for regenerative medicine in recent studies as alternatives for cell-based therapies. However, extremely low production yield, unknown effects derived from serum impurities, and relatively low bioactivities on doses must be overcome for translational applications. As several reports have demonstrated the tunability of secretion and bioactivities of EVs, herein, we introduced three-dimensional (3D) culture and cell priming approaches for MSCs in serum-free chemically defined media to exclude side effects from serum-derived impurities. Aggregates (spheroids) with 3D culture dramatically enhanced secretion of EVs about 6.7 times more than cells with two-dimensional (2D) culture, and altered surface compositions. Further modulation with cell priming with the combination of TNF-α and IFN-γ (TI) facilitated the production of EVs about 1.4 times more than cells without priming (9.4 times more than cells with 2D culture without priming), and bioactivities of EVs related to tissue regenerations. Interestingly, unlike changing 2D to 3D culture, TI priming altered internal cytokines of MSC-derived EVs. Through simulating characteristics of EVs with bioinformatics analysis, the regeneration-relative properties such as angiogenesis, wound healing, anti-inflammation, anti-apoptosis, and anti-fibrosis, for three different types of EVs were comparatively analyzed using cell-based assays. The present study demonstrated that a combinatory strategy, 3D cultures and priming MSCs in chemically defined media, provided the optimum environments to maximize secretion and regeneration-related bioactivities of MSC-derived EVs without impurities for future translational applications.

**Supplementary Information:**

The online version contains supplementary material available at 10.1186/s40580-022-00349-z.

## Introduction

Mesenchymal stem cells (MSCs) from various tissues have been employed as promising cell-based therapeutics in several diseases including myocardial disease, infection disease, sepsis, Alzheimer’s disease, and others [1, 2]. With the potential for multiple lineages differentiation of MSCs, therapeutic effects of MSCs have been known to be originated from their secretome-based paracrine signals [3]. In several types of paracrine factors, extracellular vesicle (EV) is of great interest as an important bioactive material with great therapeutic potential in multiple disease models [4]. EVs display characteristics of their parent cells and are lipid bilayer vesicles with nano-sized secretion by cells. They play an essential role in intercellular communication by transferring their compositions, proteins, miRNA, and lipids, to adjacent cells [5]. Moreover, MSC-derived EVs have been specifically studied as regenerative medicine for diseases such as wound healing [6], cardiac disease [7], neurodegeneration, [8, 9] liver fibrosis, [10] osteoarthritis [11], and renal failure [12] as an alternative to cell-based therapeutics [13]. Furthermore, EVs can be used for sepsis treatment induced by numerous factors including bacterial infections and COVID-19 [14]. EVs have been utilized as efficient therapeutics for these diseases by controlling various inflammatory factors such as IL-6, IL-8, IL-1β, TNF-α, and M1 macrophage [15–17]. Additionally, EVs are employed as a carrier of the drug for sepsis treatment with loading IκB, a potential NF-κB inhibitor [18]. Further studies are in progress to utilize EVs for rheumatoid arthritis (RA) [19], osteoarthritis (OA) [20], and chronic kidney disease (CKD) [21].

Several studies have demonstrated the potential for MSC-derived EVs in treating degeneration-related disorders. However, they are required to overcome several challenges for widespread applications, including low production yield, a large number of impurities, and low bioactivities against doses [22]. Further, there have been reports that the properties of MSC-derived EVs can be tuned with the culture environment of the cells [23, 24]. EVs from MSC with three-dimensional (3D) cell aggregates (referred to as spheroids) exhibit not only high production yield but an enhanced expression of cytokines associated with immunosuppression and anti-apoptotic properties compared to conventional two-dimensional (2D) culture [25, 26]. Moreover, it has been expected to release EVs with much higher similarity to in vivo circulating EVs due to the proximities of cell environments [27]. However, the loss of cells during the change of media is required to be improved for efficient cell culture and EV isolation from spheroids culturing media.

Research has revealed that the fate, lineage-specific differentiation, and bioactivities of MSCs can be modulated by biological and biochemical stimulations [28]. For activating MSC to increase secretion of anti-inflammatory and immunomodulatory factors, the method of preconditioning cells stimulated with proinflammatory cytokines or growth factors has been proposed as one of various priming methods [29, 30]. The preconditioning with proinflammatory cytokines contributes to produce excess amount of immunomodulatory factors to maintain homeostasis of cells. The treatment of IFN-γ, TNF-α, IL-1β, LPS, curcumin, LL37, and their combinations have been used for MSC priming [31]. Especially, our group reported the combinatory strategy of TNF-α and IFN-γ (TI) to enhance the anti-inflammatory and therapeutic properties of MSC-derived EVs for tissue regeneration [32]. Moreover, the use of fetal bovine serum (FBS) has been a controversial issue to culture MSC for isolating clinical-grade EVs due to a large number of lipoproteins and unknown membrane vesicles in FBS [33]. EV depletion methods from FBS and a process to eliminate FBS during EV isolations (starvation) have been utilized during EV isolations to eliminate the unknown side effects of vesicles in FBS [34]. However, there remain limitations due to impurities derived from remaining FBS-derived EVs and extremely low production yield of EVs in starvation conditions. To overcome these limitations, xeno-free, chemically defined, and human blood-derived replacements have been employed. We had previously suggested the serum-free chemically defined media, CellCor™ CD MSC (CDM; Xcell Therapeutics, Seoul, Korea) to isolate MSC-derived EV with high production yield and purity [35]. Moreover, a relatively large number of EVs could be isolated from healthy MSC with maintaining an excellent proliferation rate without animal-derived proteins and other impurities.

The 3D culturing and TI priming of MSCs have attracted interest to enhance the therapeutic outcome of MSC-derived EVs. However, the production yield and bioactivities related to internal cytokines of EVs have not been investigated in CDM conditions. In the present study, a comparative analysis of human umbilical cord MSCs (hUCMSC)-derived EVs from 2D and 3D culture conditions with TI priming was investigated in CDM. The characteristics of EVs derived from three different conditions (2D CDM, 3D CDM, and TI3D CDM) with optimization of the culture condition of hUCMSC with 3D spheroids and TI priming were analyzed. Further, bioinformatics analysis based on cytokine arrays of EVs was performed and expected bioactivities were confirmed by employing various in vitro cell-based assays. The comparative analyses provide optimum culture conditions and cell activation method for significant enhancement in secretion and therapeutic potential of EVs, excluding side effects derived from animal serum components for future clinical applications.

## Materials and methods

### Cell culture

Human umbilical cord mesenchymal stem cells (hUCMSCs; CHA Biotech Co. Ltd., Gyeonggi, Korea) were cultured using ɑ-MEM (NM; HyClone laboratories, UT, USA) with 10% fetal bovine serum (FBS, HyClone laboratories, UT, USA) and 1% antibiotic-antimycotic solution (GIBCO, NY, USA) or CellCor™ CD MSC media (CDM; Xcell Therapeutics, Seoul, Korea) containing 1% antibiotic-antimycotic solution for serum-free condition. hUCMSCs were seeded at the density of 3.3 × 10^3^ cells/cm^2^ for 2D and 3D cultures. To obtain spheroids of hUCMSC with 3D culture system, StemFIT 3D^®^ plate (H853400, Microfit, Gyeonggi, Korea) was utilized. After 24 h incubation in StemFIT 3D^®^ plate for spheroids formation, the spheroids were transported to the polydimethylsiloxane (PDMS)-coated flask with mounting 200 mesh Nylon filter onto the entrance and incubated for 24 h to start collecting media. For priming hUCMSC to improve regenerative potential, the combination of TNF-α and IFN-γ (TI, 20 ng/mL per each, R&D Systems, MN, USA) were pretreated to the hUCMSC for 72 h. Human proximal tubular epithelial cells (HK2; Korean Cell Line Bank, Seoul, Korea) were cultured using RPMI 1640 (HyClone laboratories, UT, USA) supplemented with 10% FBS and 1% antibiotic-antimycotic solution. Human coronary artery endothelial cells (HCAECs; Lonza, MD, USA) were cultured using endothelial growth medium-2 bullet kit (EGM-2 MV bullet kit, CC-3202, Lonza, MD, USA). All cell types were incubated at 37 °C in a humidified environment with 5% CO_2_.

### Cell viability assay

The cell viability was determined using the Cell Counting Kit-8 (CCK-8; Dojindo, Kumamoto, Japan). The CCK-8 assay was carried out in accordance with the manufacturer’s instructions to determine relative cell viability. To compare cell viability, the absorbance was measured using a microplate reader (Molecular Devices, CA, USA) at 450 nm wavelength.

### Extracellular vesicle (EV) isolation

Three types of EVs were isolated from hUCMSC culture media in different conditions, to compare therapeutic properties. To obtain EVs from 2D cultured hUCMSC in CDM (2D CDM EV), conditioned medium of hUCMSC with CDM was collected every 24 h for 4 days after stabilizing cells for 48 h. For spheroid types of hUCMSC-derived EVs without and with TI stimulation in CDM (3D CDM EV and TI3D CDM EV, respectively), conditioned media were collected for every 24 h for 4 days after spheroids formation. The collected conditioned media were centrifuged at 1,300 rpm for 3 min to remove non-exosomal large particles, such as cells, cell debris, microvesicles, and apoptotic bodies using a 0.22 μm vacuum filter/storage bottle system. After that, a filter with 500 kDa molecular weight cut-off was used in tangential flow filtration (KR2i TFF; Repligen, Waltham, MA, USA) to isolate EVs with adjusting the diafiltration rate at 7 followed by the Amicon Ultra-15 centrifugal filter (Merck Millipore, Billerica, MA, USA) to concentrate the isolated EVs.

### Characterization of EVs

The MONO ZetaView^®^ (PMX-120, Particle Metrix, Meerbusch, Germany) was used with 488 nm scatter mode to validate the size and number of EVs. Before measuring, the EV samples were diluted to 10^7^-10^8^ particles/mL with filtered phosphate-buffered saline (PBS) solution (HyClone laboratories, UT, USA). For all samples, the detailed parameters for accurate analysis were tuned with sensitivity 75, shutter 100, minimum trace length 15, and cell temperature at 25 °C. For transmission electron microscopy (TEM; Hitachi, H-7600, 80 kV, Japan) analysis to examine the EV’s morphology, The EV solution was dried on a 150-meshed formvar/carbon supported copper grid (FCF150-CU, Electron Microscopy Sciences, USA) and stained with UA-Zero (Agar Scientific, Stansted, UK) solution for a negative staining process.

### Western blot analysis

For parallel comparisons of various EVs, the same numbers of EVs (1 × 10^9^ particles) were placed onto nitrocellulose (NC) membranes after loaded with 10% sodium dodecyl sulfate-polyacrylamide gel electrophoresis (SDS-PAGE). The NC membrane was blocked using a TBST solution diluted in 5% skim milk. The EV protein-transferred NC membranes were incubated with CD81 (Santa Cruz Biotechnology, CA, USA), CD63 (Abcam, MA, USA), and CD9 (Abcam, MA, USA) primary antibodies (Abcam, MA, USA) to confirm surface markers of EV, and N-cadherin (13,116 S, 1000:1, Cell Signaling Technology, MA, USA), fibronectin (ab2413, 500:1, Abcam, MA, USA), and GAPDH (5174T, 1000:1, Cell Signaling Technology, MA, USA) primary antibodies to demonstrate anti-fibrotic properties of EV, respectively, followed by incubation with HRP-linked secondary antibodies (Cell Signaling Technology, MA, USA). The blot was pretreated with the enhanced chemiluminescence solution (GE Healthcare, WI, USA) and visualized with ChemiDoc™ XRS + and ImageLab software (Bio-Rad, CA, USA).

### Fourier transform infrared (FTIR) measurement

All EV samples were dissolved in PBS solution for FTIR measurement with the Spectrum Two FTIR spectrometer (PerkinElmer, CT, USA) in transmission mode. EVs were deposited onto a calcium fluoride (CaF_2_) window and dried for 10 min under a nitrogen stream. All FTIR spectra of EVs were obtained in the 900–4000 cm^− 1^ wavenumber region, with a spectral resolution of 4 cm^− 1^ and 32 scans. A background scan was validated using a CaF_2_ window filled with PBS solution.

### 3D principal component analysis (3D-PCA)

The spectra of FTIR between 2800 and 3100 and 900–1880 cm^− 1^, were used for the principal component analysis (PCA) of EVs. Min-max normalization was utilized to normalize the selected data and the data were centered with the mean subtraction approach. Principal component analysis was used to get PCA for spectroscopic applications with OriginPro 2017 software (OriginLab, MA, USA). PC1, PC2, and PC3 were utilized to represent the location of each element in a 3D graph.

### Cytokine array

The radio-immunoprecipitation assay (RIPA; Rockland Immunochemicals, PA, USA) buffer was used to lyse the same numbers of EVs (1 × 10^10^ particles). The lysed EV solutions were placed onto the NC membrane of the Proteome Profiler™ Antibody Arrays Human XL Cytokine Array Kit (R&D Systems, MN, USA). After developing the NC membrane with ChemiDoc™ XRS+, the intensities of the cytokine array were quantified using ImageLab software. For data analysis, the average intensity of cytokine array was exported with expression of cytokine array intensities in logarithm base 2. The PANTHER (Protein Analysis THrough Evolutionary Relationships; http://www.pantherdb.org) was utilized to analyze the representative proteins from the cytokine array. The gene ontology (GO) and KEGG pathway were analyzed using the DAVID (Database for annotation, visualization and integrated discovery; https://david.ncifcrf.gov/) for prediction of functionalities.

### Enzyme-linked immunosorbent assay (ELISA)

The Quantikine™ ELISA kit (R&D Systems, MN, USA) was used to validate angiogenic factors (HGF and bFGF) in EVs. The equal numbers of EVs (1 × 10^9^ particles) were loaded into ELISA wells, and ELISA procedure was carried out in accordance with the manufacturer’s instructions. The absorbance was measured at 450 nm using a microplate reader and subtracts were measured at wavelengths of 540 and 570 nm, respectively.

### Tube formation assay

On 24 well plates (Corning, NY, USA) coated with Matrigel, 1 × 10^5^ cells/well of HCAECs were cultured for 18 h. The same concentration of EVs (1 × 10^8^ particles/mL) from three types of conditioned media (2D CDM, 3D CDM, TI 3D CDM) were treated to the HK2 cells. Calcein AM stained cells were visualized to monitor tube formation using fluorescence microscopy (CKX53, OLYMPUS, Japan). The ImageJ (Wayne Rasband, NIH, USA) plugin for the angiogenesis analyzer was employed to quantify angiogenesis-related properties.

### Wound healing assay

The HK2 cells were cultivated until confluent with a monolayer at the density of 2 × 10^5^ cells per well in 6 well plates. A sterilized 1 mL pipette tip was used to scratch the cells on the center of the wells followed by the treatment with same concentration of EVs (1 × 10^8^ particles/mL) from three different conditioned media (2D CDM, 3D CDM, and TI3D CDM) after rinsed with PBS solutions. A microscopy was used to visualize the cell migration after 24 h incubation. The ImageJ (Wayne Rasband, NIH, USA) plugin for the wound healing tool was utilized to assess the percentage of open area.

### Immunocytochemistry (ICC)

The HK2 cells were fixed in 4% paraformaldehyde at room temperature for 20 min and 0.2% Triton-x diluted in PBS solution was used to increase cell permeability for 10 min at the same temperature. Then, it was blocked with 1% BSA dissolved in PBS solutions, followed by being incubated overnight at 4 °C with the primary antibody then incubated 1 h at room temperature in dark with the secondary antibody. The various antibodies, NF-κB primary antibody (SC-8008, 2 µg/mL, Santa Cruz Biotechnology, CA, USA) for inflammatory markers, fibronectin primary antibody (ab2413, 500:1, Abcam, MA, USA) for fibrosis marker, and donkey anti-rabbit IgG (H + L) highly cross-adsorbed secondary antibody, Alexa Fluor™ 488 (A-21,206, 200:1, Invitrogen, CA, USA) for immunofluorescent labeling, respectively, were used. The nucleus was stained with Hoechst (62,249, 1 µg/mL, Thermo Fisher Scientific, OH, USA), and immunofluorescence images were obtained using a fluorescence microscopy (CKX53, OLYMPUS, Japan).

### RT-qPCR for pro-inflammatory factors

The HK2 cells were seeded at the density of 2 × 10^5^ cells/well on 6 well plates. Both TNF-α and the EV were treated for 24 h simultaneously. After 24 h, RNA was extracted for real time quantitative PCR (RT-qPCR) to determine the level of inflammation. The SYBR green PCR reagent mix (Applied Biosystems, CA, USA) was applied for RT-qPCR. The QuantStudio 3 (Applied Biosystems, CA, USA) was used to initiate reactions with the following primers. NF-κB: forward, 5′-cgggatggcttctatgagg-3′, and reverse, 5′-ctccaggtcccgcttctt-3′; IL-6: forward, 5′-gatgagtacaaaagtcctgatcca-3′ and reverse, 5′-ctgcagccactggttctgt-3′; IL-8: forward, 5′-agacagcagagcacacaagc-3′ and reverse, 5′-atggttccttccggtggt-3′; 18 s rRNA: forward, 5′- gcaattattccccatgaacg-3′ and reverse, 5′-gggacttaatcaacgcaagc-3′. The data were quantified using 2-ΔΔCt method with 18 s rRNA as a reference.

### Apoptosis assay

After HK2 cells were seeded at 2 × 10^5^ cells/well on 6 well plates, the 0.5 mM of hydrogen peroxide (H_2_O_2_) was treated 2 h after EV treatments to cells. To measure apoptosis rates with flow cytometry, FITC Annexin V Apoptosis Detection Kit I (556,547, BD Biosciences, CA, USA BD Biosciences) was used as manufacturer’s instructions. After labeling cells, flow cytometry (CytoFLEX; Beckman Coulter, CA, USA) was employed to classify labeled apoptotic cells.

### Fibrosis assay

After 24 h treatment of TGF-β (2 ng/mL) onto the HK2 cells, seeded at the density of 2 × 10^5^ cells/well in 6 well plates, same concentration of three types of EVs (1 × 10^8^ particles/mL) were treated to the cell for 24 h. Cell nucleus and fibronectin were stained using Hoechst and fibronectin primary antibody for ICC, and proteins were extracted to compare inhibition activity of EVs for fibrosis markers, N-cadherin and fibronectin, using Western blot analysis.

### Statistical analysis

GraphPad Prism 7 was used to assess every statistical analysis (GraphPad Software, CA, USA). Unpaired *t* tests or one-way analysis of variance (ANOVA) with Tukey’s multiple comparison post-test were assessed to distinguish group differences. *p* values below 0.05 were determined statistically significant (*p < 0.05; **p < 0.01; ***p < 0.001; ****p < 0.0001).

## Results and discussion

### 3D culture system for EV isolation

**Fig. 1:**
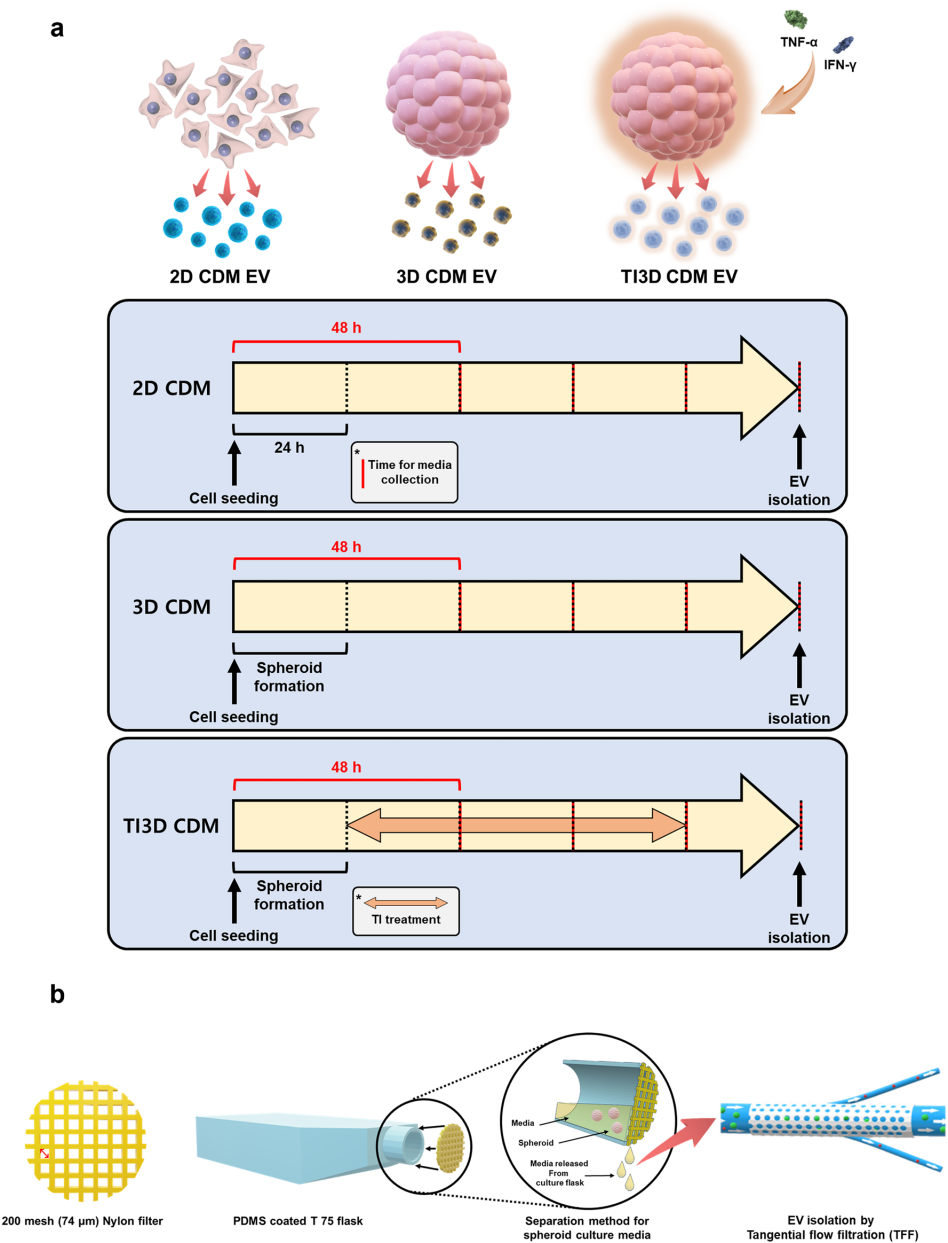
The schematic representation for human umbilical cord mesenchymal stem cells (hUCMSC)-derived extracellular vesicles (EVs) with 3D culture and TI priming in chemically defined media (CDM). **a** Time schedules for cell culture and EV isolation. **b** A set up for media changeable 3D spheroid culture system

**Fig. 2:**
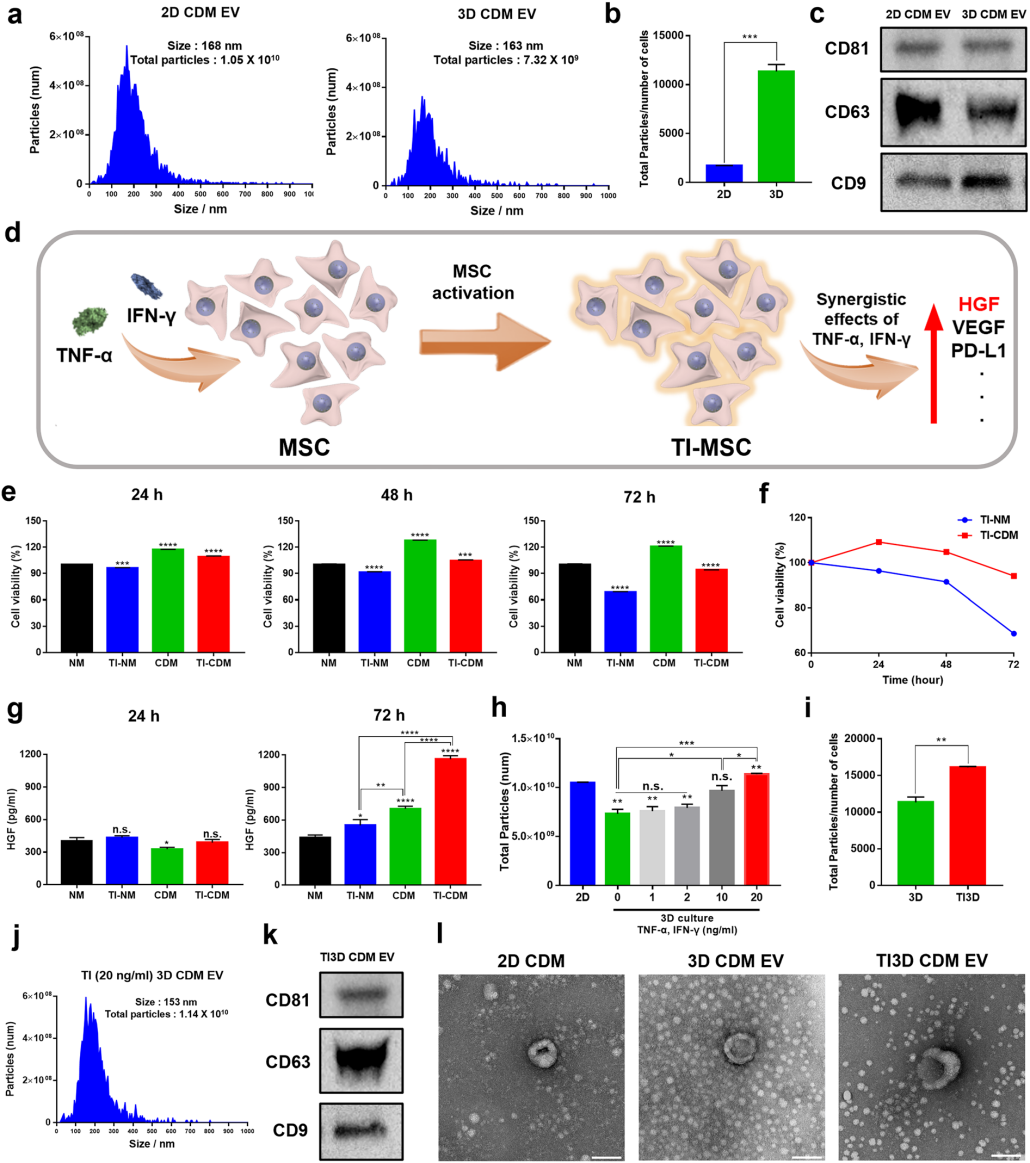
Characterizations of EV from three different culture conditions.** a** ZetaView^®^ analysis for the size and number of total particles of 2D CDM EV and 3D CDM EV. **b** The number of particles per cell for 2D CDM EV and 3D CDM EV. **c** The Western blot analysis of 2D CDM EV and 3D CDM EV for representative surface markers of EVs. **d** The benefits of MSC activation using TNF-α and IFN-γ (TI) priming. **e** Time-dependent cell viability with TI activation in NM and CDM (NM; Normal media, 10% fetal bovine serum supplemented ɑ-MEM, CDM; CellCor™ CD MSC media). **f** Comparison of cell viabilities in TI-activated hUCMSC in NM and CDM. **g** The expression level of HGF in culture media of hUCMSC. **h** The total number of particles derived from different concentrations of TI treatments for hUCMSC spheroids in CDM. **i** The number of particles per cell with and without TI priming in 3D culture. **j** The size and total number of particles for TI3D CDM EV were analyzed using ZetaView^®^. **k** The Western blot analysis for representative surface markers of TI3D CDM EV. **l** The morphologies of EVs are characterized by TEM. Scale bars equal to 100 nm. (Values are presented as mean ± SD (n = 3) and statistical significance was obtained with one-way analysis of ANOVA with Tukey’s multiple comparison post-test (*p < 0.05; **p < 0.01; ***p < 0.001; ****p < 0.0001))

Figure 1a illustrates the time schedules for culturing hUCMSC and isolating EVs to compare the secretion and functionalities of hUCMSC-derived EVs. In a 2D system cultured with CDM (2D CDM), the EVs were isolated every 24 h for 4 days. In a 3D culture system with CDM (3D CDM), the EVs were isolated after spheroid formation every 24 h for 4 days (Fig. 1a). Further, before culturing spheroids for EVs isolation, spheroids were formed in StemFIT 3D^®^ plate for 24 h (Additional file 1: Fig. S2a) and incubated additional 24 h with transferring to PDMS-coated culture plate. The total period of collecting cell media for EV isolation was set equal to 4 days for parallel comparisons. The more frequent collection of conditioned media can be expected to increase the production yield of EVs [36]. Moreover, the culturing process of spheroids has been widely thought to improve the secretion and functionalities of EVs [37, 38]. However, the limitation of difficulties in changing culture media has to be overcome during spheroid culture for sequential collecting media [39]. In this study, we established a novel system that can be employed to change the culture media in the spheroid culture system (Fig. 1b). A 76 μm Nylon mesh was mounted onto the entrance of a 75T flask which inhibited the loss of spheroids (the size ~ 100 μm) during the collection of media for EV isolations. The EVs were successfully isolated using the tangential flow filtration system, and the size and number of EVs were analyzed with the MONO ZetaView^®^. Figure 2a displays the size and number of particles, EVs (168 nm, 1.05 × 10^10^ particles) from 2D CDM (2D CDM EV) and EVs (163 nm, 7.32 × 10^9^ particles) from 3D CDM (3D CDM EV), respectively. Culturing UCMSCs in CDM and serial filtration with 0.22 μm vacuum filter/storage bottle and 500 kDa cutoff filter of TFF system during EV isolations enable to increase the purity of EVs [34, 35]. The variation in the number of EVs could be due to the differences in rates of cell proliferation between 2D and 3D culture systems using CDM. Our previous results demonstrated that CDM accelerated the proliferation rate of hUCMSC in 2D culture conditions. The same number of cells were seeded in both 2D and 3D culture systems and the numbers of cells on the 5 days after cell seeding were 6.1 × 10^6^ and 6.5 × 10^5^ in 2D CDM and 3D CDM, respectively. The number of cells was estimated with variation in the average diameter and volume of the spheroids after 5 days in 3D CDM (Additional file 1: Figs. S1, S2b, c). Furthermore, the number of particles per cell was calculated to compare the efficiency of EV secretion accurately. The number of particles per cell displayed a ~ 6.7 times increase in 3D culture conditions than in 2D (Fig. 2b). The representative EV markers (CD81, CD63, and CD9) were validated using Western blot analysis as the guideline of MISEV 2018 for further verification (Fig. 2c) [40]. Furthermore, the secretion of hUCMSC-derived EVs could be enhanced with the 3D culturing system in CDM as demonstrated in conditions for a serum-containing media.

### TNF-α /IFN-ɣ primed hUCMSC-derived EVs in CDM condition

Combined with 3D culture of hUCMSCs to promote secretion of EVs, TNF-α/IFN-ɣ (TI) priming was additionally applied to 3D spheroids hUCMSCs in CDM (TI3D CDM) to stimulate hUCMSC for boosting EV secretion and functionalities during 3D culture in CDM. Figure 1a shows that the spheroids of hUCMSC were activated with TI for 3 days. To date, a lot of studies have shown that TI priming upregulates anti-inflammatory and immunosuppressive properties of MSCs under 10% FBS supplemented media conditions [28, 41]. The activated MSC by TI priming (TI-MSC) produces more EVs and cytokines such as HGF, VEGF, PD-L1, and others (Fig. 2d). Moreover, the TI priming process has been known to reduce cell viability, especially in serum-free conditions for EVs isolations [42]. Further, to optimize TI priming conditions for hUCMSC cultured in CDM, the cell viability was compared in two different media conditions i.e., 10% FBS supplemented ɑ-MEM (Normal media; NM) and serum-free CDM. During TI priming, the cell viability decreased by about 68% and 94% in NM (TI-NM) and CDM (TI-CDM) conditions, respectively (Fig. 2e and f). The maximum concentration of TI was set at 20 ng/mL to minimize cellular toxicity [32, 41, 42]. Compared to NM, the CDM condition enabled hUCMSC to viable during the TI priming process. This proves the advantages of isolating EVs from healthy cells even in the TI stimulations as well as being able to isolate EVs under serum-free conditions. Furthermore, to confirm TI priming-based activation of hUCMSC, the level of HGF, which is known to be upregulated in activated MSCs, was evaluated using ELISA. The concentrations of HGF were elevated both in NM and CDM after 48 h of TI treatments. Figure 2g displays that HGF was released approximately two times more in TI-activated hUCMSC in CDM compared to NM. These results indicated that TI-activated hUCMSC-derived EVs in CDM (TI3D CDM EV) have a higher potential for maturation of endothelial and vascular cells due to upregulating level of proangiogenic factors. Moreover, the limitation of the relatively low number of total EVs in 3D CDM EV than a large number of cells with a strong proliferation rate of 2D cultured hUCMSC in CDM could be overcome with TI stimulations at the concentration of 20 ng/mL. The size of particles was similar (153 nm) and total number of particles increased 1.5 times in 3D conditions with TI activation (TI 3D CDM) compared to conditions for 3D CDM (Fig. 2h). The number of particles per cell improved about 1.4 times with TI activation in 3D CDM (Fig. 2i). Finally, 1.14 × 10^10^ particles with optimizing conditions for TI primed spheroids in CDM could be isolated (Fig. 2j). Furthermore, the surface markers of EVs, CD81, CD63, and CD9, were evaluated via Western blot analysis (Fig. 2k). Moreover, the morphologies of vesicles were also investigated using TEM (Fig. 2L). Three types of EVs from different culture conditions i.e., 2D CDM EV, 3D CDM EV, and TI3D CDM EV displayed similar morphologies and expressions of surface markers [43].

### The comparative analysis of EV’s internal and external properties

**Fig. 3:**
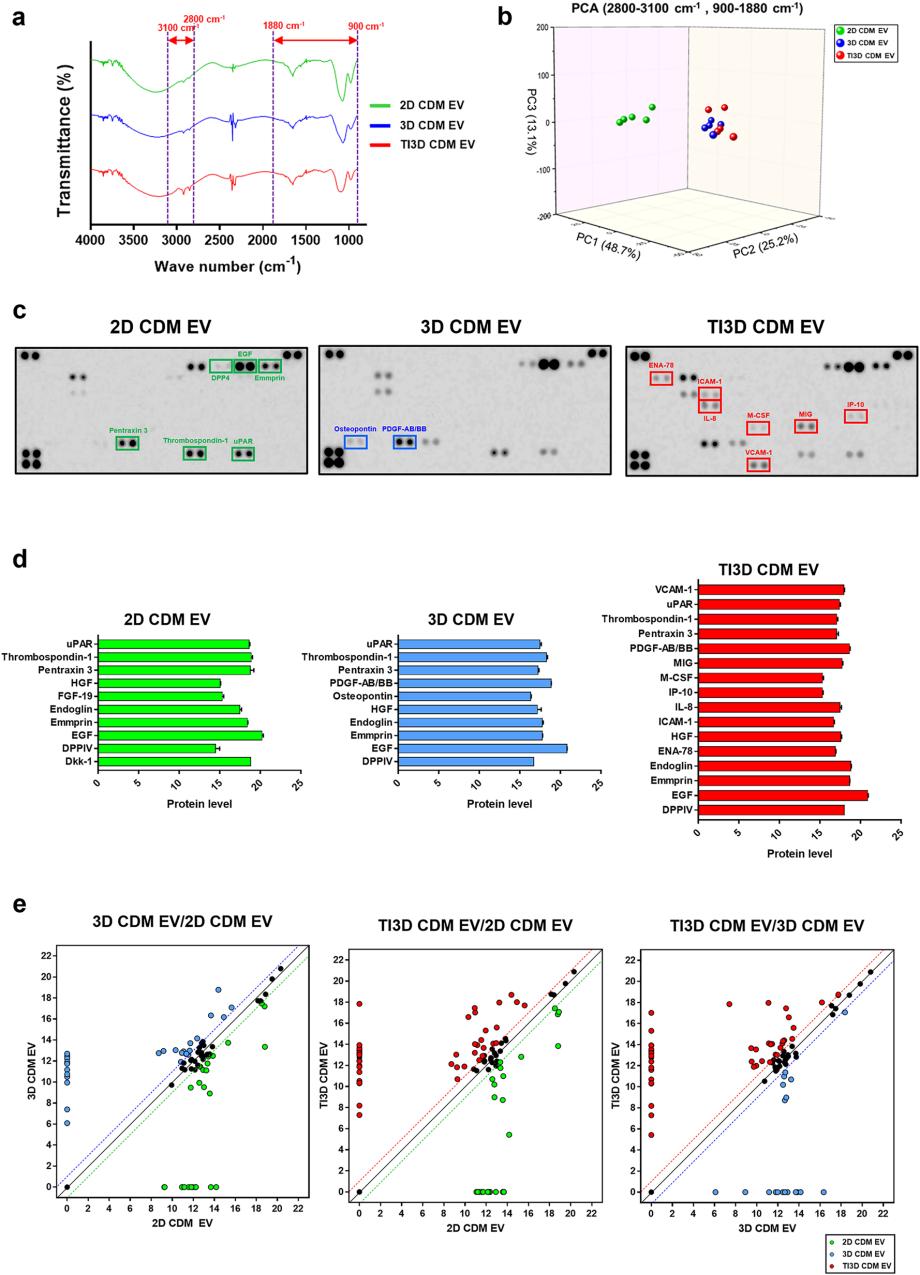
Internal and external characteristics of EVs.** a** The FTIR spectra of EVs in the range of 900–4000 cm^−1^ wavelength to distinguish the surface compositions of EVs. **b** The 3D PCA score plot to classify EVs from FTIR analysis. **c** The representative images of cytokine array membranes of 2D CDM EV, 3D CDM EV, and TI3D CDM EV. **d** The comparison of expression levels for representative cytokines. **e** The scatter plot of three different EVs by the results of cytokine arrays

The FTIR was performed to distinguish subpopulations of EVs based on structural changes of surface compositions on EVs such as proteins and lipids, depending on the differences in culture conditions (2D CDM, 3D CDM, and TI3D CDM). Figure 3a shows the FTIR spectra, with peaks at 1650 and 1540 cm^−1^ for amide I and II absorption, 3050–2800 and 1500–1350 cm^−1^ for lipid acyl chain absorption, and 1740 cm^− 1^ for ester carbonyl group absorption. The peaks were monitored to classify the subpopulation of EVs from different culture conditions (Fig. 3a) [44]. The principal component analysis (PCA) is a statistical method used to apply collective analysis from various parameters [45]. The differences in FTIR spectra mean different surface compositions, hence EVs were classified and visualized with PCA in three-dimensional (3D) spaces. Interestingly, PCA results exhibited a clear separation between 2D CDM EV and 3D CDM EV, whereas they showed similar distribution between 3D CDM EV and TI3D CDM EV (Fig. 3b). The results speculated that the external components of EVs could be changed by 3D culture compared to 2D culture, and TI priming did not induce changes in the external composition of EVs.

The EVs are known to exhibit the characteristics of parent cells by paracrine effects. The internal components of EV were analyzed using a cytokine array method to verify the dependence of functionalities of EV on cell culture conditions and the TI priming process (Fig. 3c). The TI3D CDM EV expressed more regeneration-related factors than the 2D and 3D CDM EVs. The cytokine expressions for three different kinds of EVs were analyzed. The representative cytokines were selected from the cytokine array and the relative expression level was quantified from intensities on membranes of the cytokine array using ImageLab software (Fig. 3d). Our previous study demonstrated that the regeneration-related factors, including dipeptidyl peptidase 4 (DPP-4), epidermal growth factor, emmprin, thrombospondin-1, urokinase-type plasminogen activator receptor (uPAR), and pentraxin-3 were highly expressed in EVs from hUCMSC cultured in CDM (2D CDM EV) [46]. Osteopontin (OPN) and platelet-derived growth factor AB/BB (PDGF-AB/BB) were newly emerged in EVs (3D CDM EV) with culturing in 3D conditions. In particular, PDGF-AB/BB is a well-known regenerative factor that promotes wound healing, cell proliferation, and bone regeneration [47]. Furthermore, compared to changing the culture condition from 2D to 3D, activating hUCMSC with TI priming highly enhances EVs with regeneration-relative cytokines, including epithelial-derived neutrophil-activating peptide-78 (ENA-78), intercellular adhesion molecule-1 (ICAM-1), interleukin-8 (IL-8), macrophage colony-stimulating factor (M-CSF), vascular cell adhesion molecule-1 (VCAM-1), monokine induced by gamma interferon (MIG), and interferon gamma-induced protein-10 (IP-10). Moreover, TI priming has been known to upregulate chemokine ligands (IP-10), adhesion proteins (ICAM-1 and VCAM-1), and anti-inflammatory cytokine (IL-8) levels. Further, angiogenesis-related cytokine (ENA-78) [48] and macrophage stimulating protein (M-CSF) [49] are highly expressed in TI primed hUCMSC-derived EVs, which promote tissue regeneration. The scatter plot based on the cytokine array to compare the differences in internal cytokines between 2D CDM EV and 3D CDM EV, 2D CDM EV and TI3D CDM EV, and 3D CDM EV and TI3D CDM EV, respectively (Fig. 3e). Contrary to FTIR results, TI priming induced differences in cytokine expressions compared to changing culture condition with 3D, suggesting that TI priming activates hUCMSCs and alters internal cytokine components.

### Expectation for the functionality of UCMSC-derived EVs based on bioinformatics analysis

**Fig. 4:**
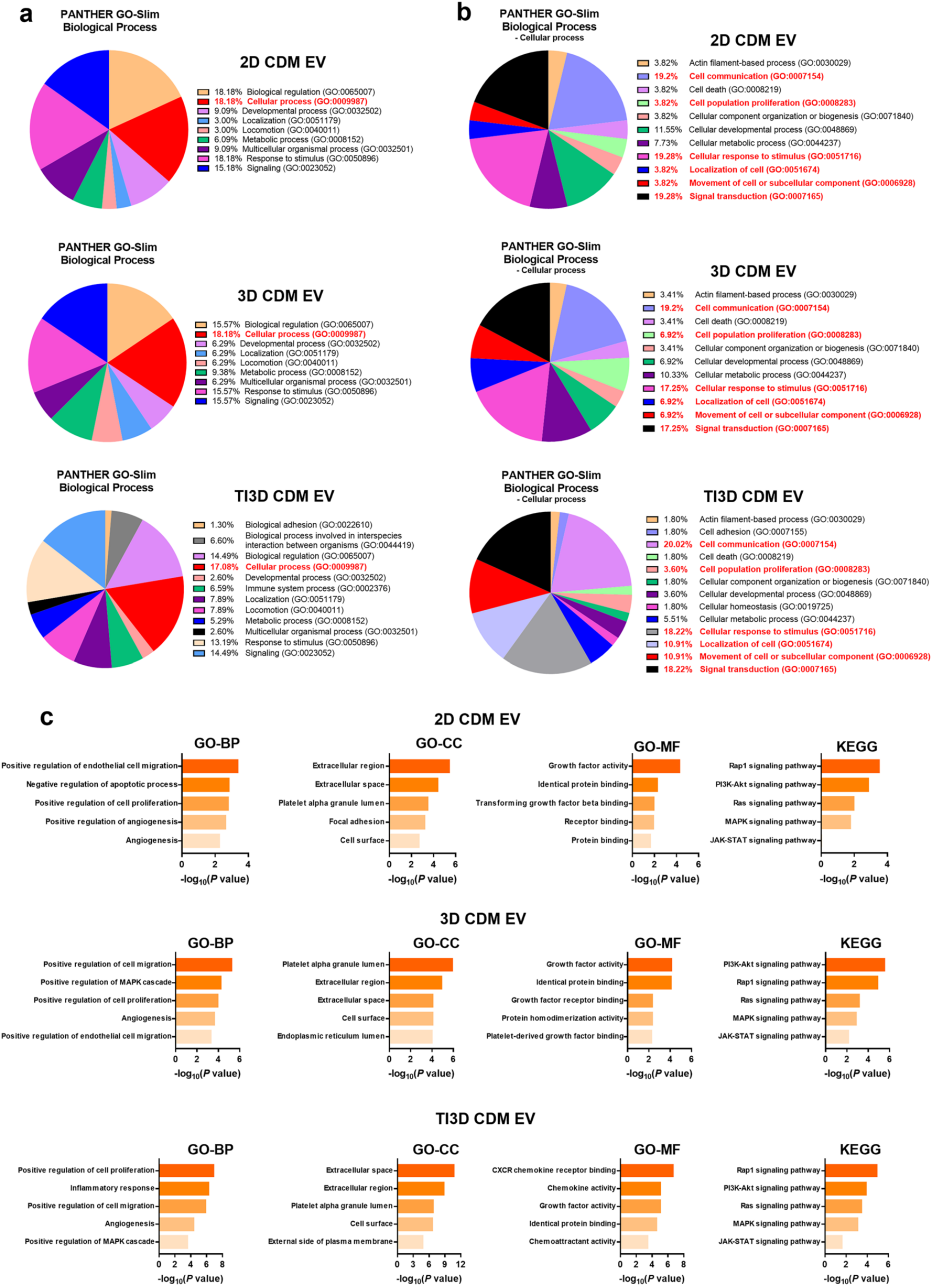
Bioinformatics analysis of EVs.** a** The PANTHER analysis for highly expressed factors of three types of EVs. **b** The DAVID analysis for Gene Ontology (GO)-BP, GO-CC, GO-MF, and Kyoto Encyclopedia of Genes and Genomes (KEGG) pathway

The protein analysis through evolutionary relationships (PANTHER) to anticipate the therapeutic potentials of EVs based on internal components was performed. PANTHER integrates genomes, gene function classifications, pathways, and statistical analysis tools to help researchers analyze huge quantities of genome-wide experimental data [50]. Figure 4a shows that the cellular processes constitute the largest cluster of biological processes in all EV groups. Among the clusters of cellular processes, angiogenesis and wound healing-related properties, including cell communication, cell population proliferation, cellular response to stimulus, localization of cell, movement of a cell or subcellular component, and signal transduction, were highlighted and they made up large parts of cellular processes (Fig. 4b). The sum of these clusters showed that TI3D CDM EV had the highest value (81.88%) compared to 2D CDM EV (69.22%) and 3D CDM EV (74.46%). These results indicate that the TI3D CDM EV could promote angiogenesis and wound healing effects for regeneration.

Further, the various modules of the database for annotation, visualization, and integrated discovery (DAVID) were utilized to anticipate cellular responses for three types of EVs. The biological processes (BP), cellular components (CC), molecular functions (MF), and signaling pathways regulated by the protein selected on the cytokine array were investigated using Gene Ontology (GO) and the Kyoto Encyclopedia of Genes and Genomes (KEGG) in DAVID. Figure 4c depicts the top 5 GO-BP enrichments in three types of EVs including regeneration-related properties such as cell migration, cell proliferation, anti-apoptosis, angiogenesis, and inflammatory response. Moreover, the DAVID was conducted with the data of the EV cytokine array. The largely represented GO-CC terms were “Extracellular region” and “Extracellular space,” indicating the origin of the EV from the cell. In GO-MF terms, “Growth factor activity” and “Identical protein binding” showed relatively large *p* values for all CDM EVs. Further, “growth factor activity” is related to the activity that prompts cell growth and proliferation. The hUCMSC-derived EVs in CDM (2D CDM EV, 3D CDM EV, and TI3D CDM EV) exhibited a high correlation with “growth factor activity,” which can facilitate cell proliferation as expected by results of GO-BP. Interestingly, the GO-MF results from the TI3D group displayed a high *p* value for “CXCR chemokine receptor binding” and “chemokine activity.” The chemokines are recognized as significant chemotactic chemicals that control the trafficking and function of immune cells. Further, it is expected to elevate the immunomodulatory properties of TI3D CDM EV compared to other groups. The results of KEGG analysis implied that hUCMSC-derived EVs from three different culture conditions were significantly involved in regenerative signaling pathways, such as the “Rap1 signaling pathway,” “PI3K-Akt signaling pathway,” “Ras signaling pathway,” “Mitogen-activated protein kinase signaling pathway,” and “JAK-STAT signaling pathway.” The Rap1 signaling pathway is well-known to relate to cell adhesion and angiogenesis [51, 52]. The PI3K-AKT signaling pathway mediates lots of biological activities such as cell division, autophagy, survival, differentiation, and bone formation [53]. Additionally, the Ras signaling pathway was related to cell proliferation and differentiation [54]. Furthermore, based on DAVID analysis, it was hypothesized that all kinds of hUCMSC-derived EVs (2D CDM EV, 3D CDM EV, and TI3D CDM EV) can be expected to facilitate angiogenesis, wound healing, anti-inflammation, and anti-apoptosis for tissue regenerations. The differences in biological activity should be compared with cell-based assays based on the results of a bioinformatics analysis.

### Angiogenesis and wound healing effects of EVs

**Fig. 5:**
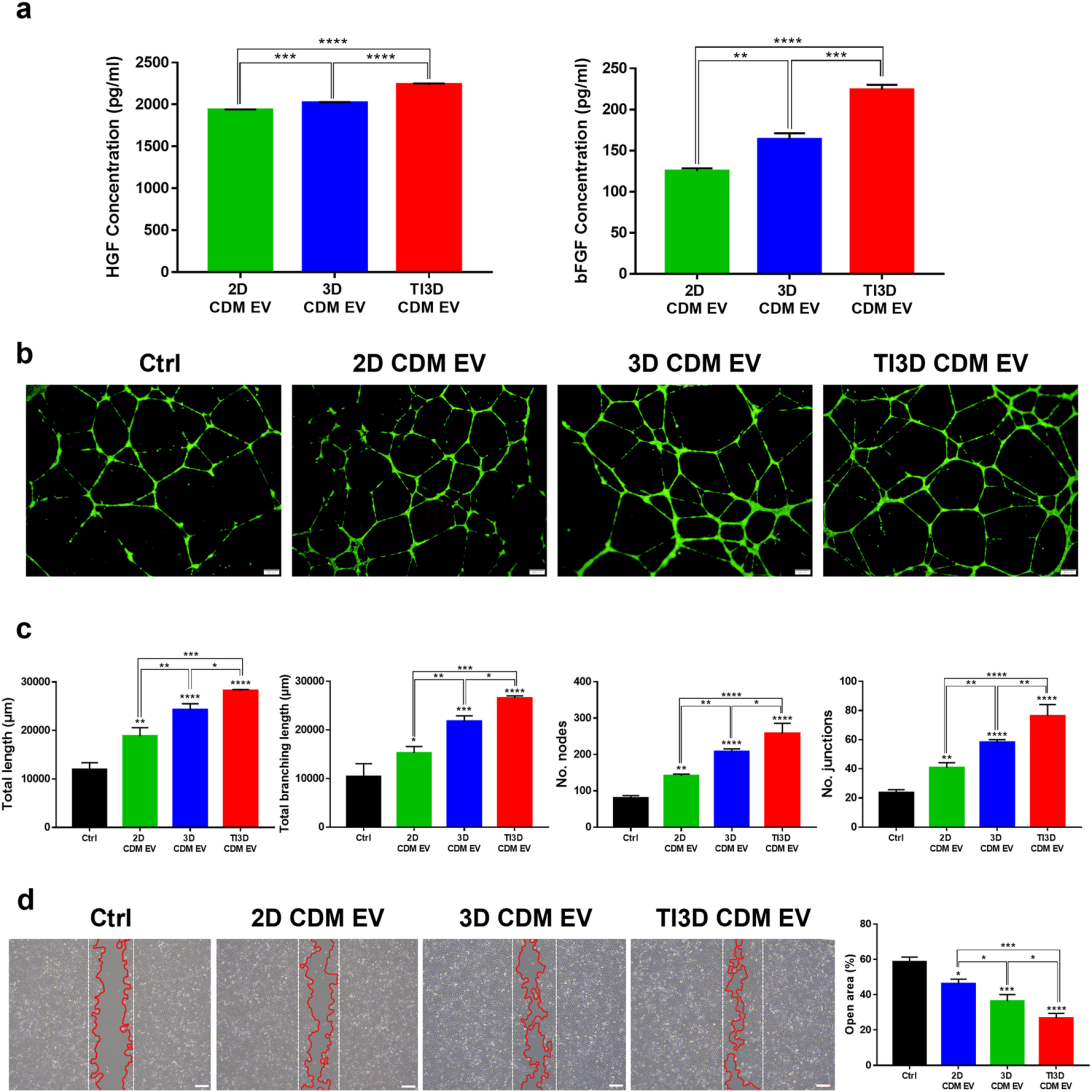
Angiogenesis and wound healing effects of EVs.** a** The HGF and bFGF levels of EVs. **b** Representative images of angiogenesis effect by tube formation assay of EVs. **c** Analysis of total length, total branching length, number of nodes, and number of junctions from tube formation assay. **d** The representative images of cell migration assays to evaluate wound healing effects of EVs, and calculated wound healing rates of EVs. (Analysis with Image J; Values are presented as mean ± SD (n = 3) and statistical significance was obtained with one-way analysis of ANOVA with Tukey’s multiple comparison post-test (*p < 0.05; **p < 0.01; ***p < 0.001; ****p < 0.0001))

The hUCMSC-derived EVs were expected to contain angiogenesis-related factors from the bioinformatics analyses. Figure 5a shows the expression levels of HGF and bFGF validated with ELISA. The expression levels of HGF and bFGF were observed to be greater in TI3D CDM EV, with TI priming in 3D culture condition, than in the other groups. HGF has an angiogenic effect and the potential to stimulate cell motility or invasion which promotes cell proliferation and resists apoptosis [55]. In addition, bFGF has a strong mitogenic effect on vascular and capillary endothelial cells, and it can also promote angiogenesis for tissue regenerations [56]. Further, to demonstrate the strong angiogenic property of TI3D CDM EVs, the angiogenesis-related tube formation assay was performed with HCAEC (Fig. 5b). The results showed that the ability of tube formation was significantly accelerated in the TI3D CDM EV. Moreover, various angiogenic-related parameters such as total length, total branching length, number of nodes, and number of junctions, were quantified to verify the effects of EVs for tube formation at the indicated time in many ways (Fig. 5c). All parameters of tube formation exhibited gradual increase with the introduction of 3D culture and TI priming processes. A robust and vigorous angiogenic response is required for wound healing due to the requirement of oxygen and nutrients for cellular proliferation and migration and metabolic processes [57]. Further, to compare wound healing properties for three types of EVs, wound closure rates of scratch formed HK2 cells were monitored 24 h after incubating EVs. The migration rate of HK2 cells increased at all kinds of EVs compared to cells without EVs treatments at the same trends with tube formation activity (Fig. 5d). Figure 3 shows the strong tube formation and wound healing abilities of TI3D CDM EV are correlated with the results of cytokine array. Specifically, a higher expression level of ENA-78 and PDGF-AB/BB could facilitate angiogenic properties, and that of cell adhesion proteins, ICAM-1 and VCAM-1, could enhance cell migration activities of TI3D CDM EV [58].

### Anti-inflammatory effects of EVs

**Fig. 6:**
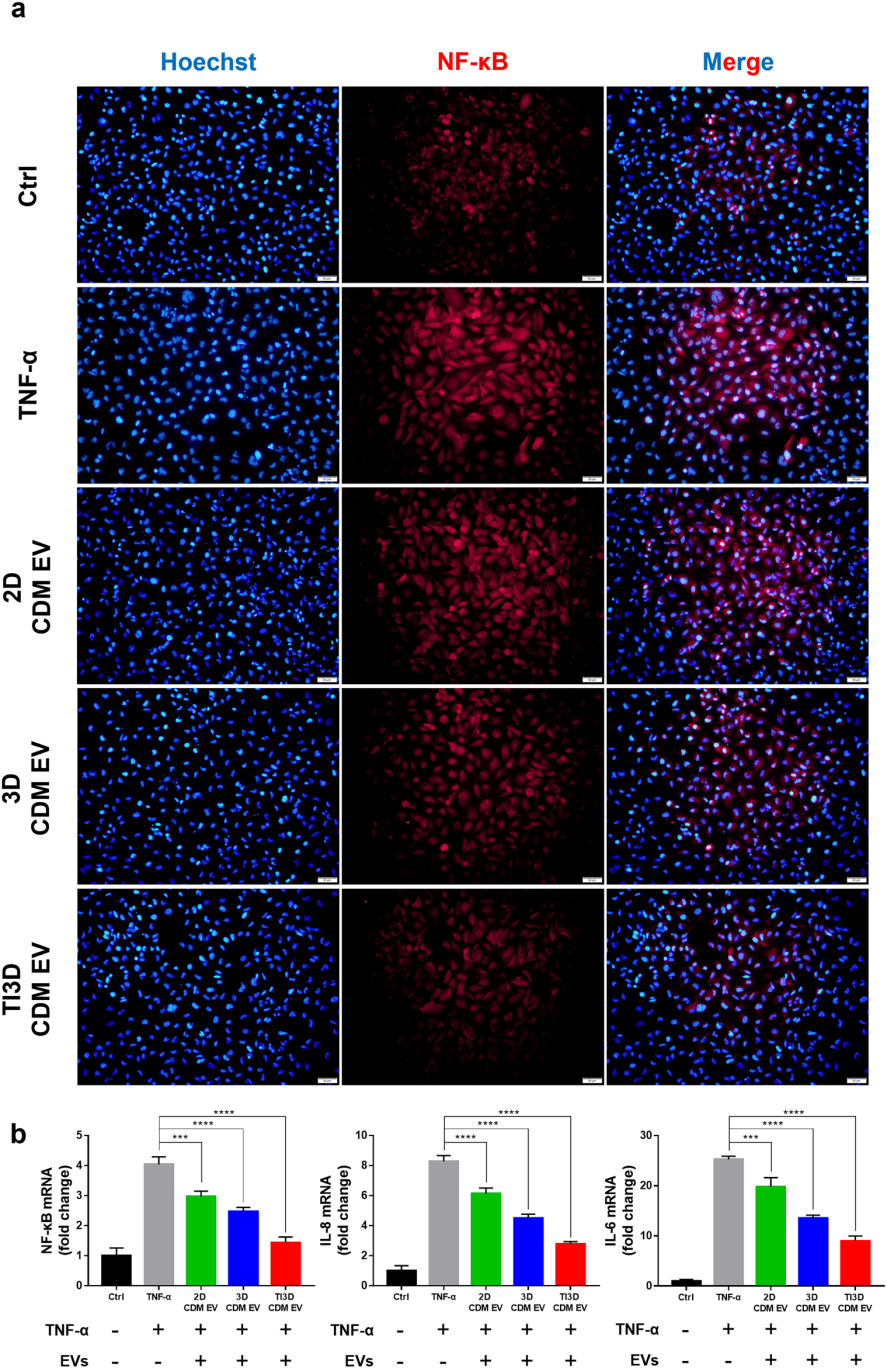
Anti-inflammatory activities of EVs. **a** The fluorescence-based immunocytochemistry for NF-κB expression of TNF-α induced inflammation model with HK2 cells. **b** The gene expression levels of representative inflammatory factors, NF-κB, IL-8, and IL-6

The inflammation is associated with a variety of diseases, including sepsis which can be followed by COVID-19, chronic obstructive pulmonary disease, chronic obstructive pulmonary disease (COPD), CKD, and others [59]. MSC-derived EVs have been known to show immunomodulatory and anti-inflammatory effects on inflammatory tissues [60]. Further, to investigate the anti-inflammatory effects of EVs, the changes in expression level for the major inflammatory factor, NF-κB, were monitored in TNF-α stimulated HK2 cells using immunocytochemistry (ICC) (Fig. 6a). Moreover, after TNF-α treatment for acute inflammation, the level of NF-κB dramatically increased. However, the levels were slightly attenuated in the 2D CDM EV group and demonstrated more downregulation in 3D CDM EV. The differences in internal cytokines of EVs decided by the culture condition of the parent cell, 2D and 3D, could subsequently impact the anti-inflammatory properties as described in the cytokine array and bioinformatics analysis results. Additionally, TI3D CDM EV treated cells released significantly low amounts of NF-κB compared to other groups as expected. TI priming can activate MSCs to release anti-inflammatory factors to restore themselves from proinflammatory cytokine stimulus [61]. Further, the results of quantitative reverse transcription PCR (RT-qPCR) disclosed that TI3D CDM EV downregulated the expression of NF-κB the most in comparison with the 2D CDM EV and 3D CDM EV as the same trends with ICC results (Fig. 6b). Interestingly, the gene level of NF-κB showed similar with the control group in TI3D CDM EV treated group. Results from RT-qPCR revealed that IL-8 and IL-6 were also downregulated. NF-κB plays a critical role in immune modulation, angiogenesis, cell proliferation, apoptosis, and inflammatory response [62, 63]. Particularly, the NF-κB is widely accepted as a major contributor to inflammatory disorders [64] and stimulates the release of IL-8 and IL-6 during inflammatory responses [65]. Moreover, the RT-qPCR results demonstrated that TI3D CDM EV displayed strong anti-inflammatory activities via suppressing the major inflammation mediator, NF-κB.

### Anti-apoptosis effect of EVs

**Fig. 7:**
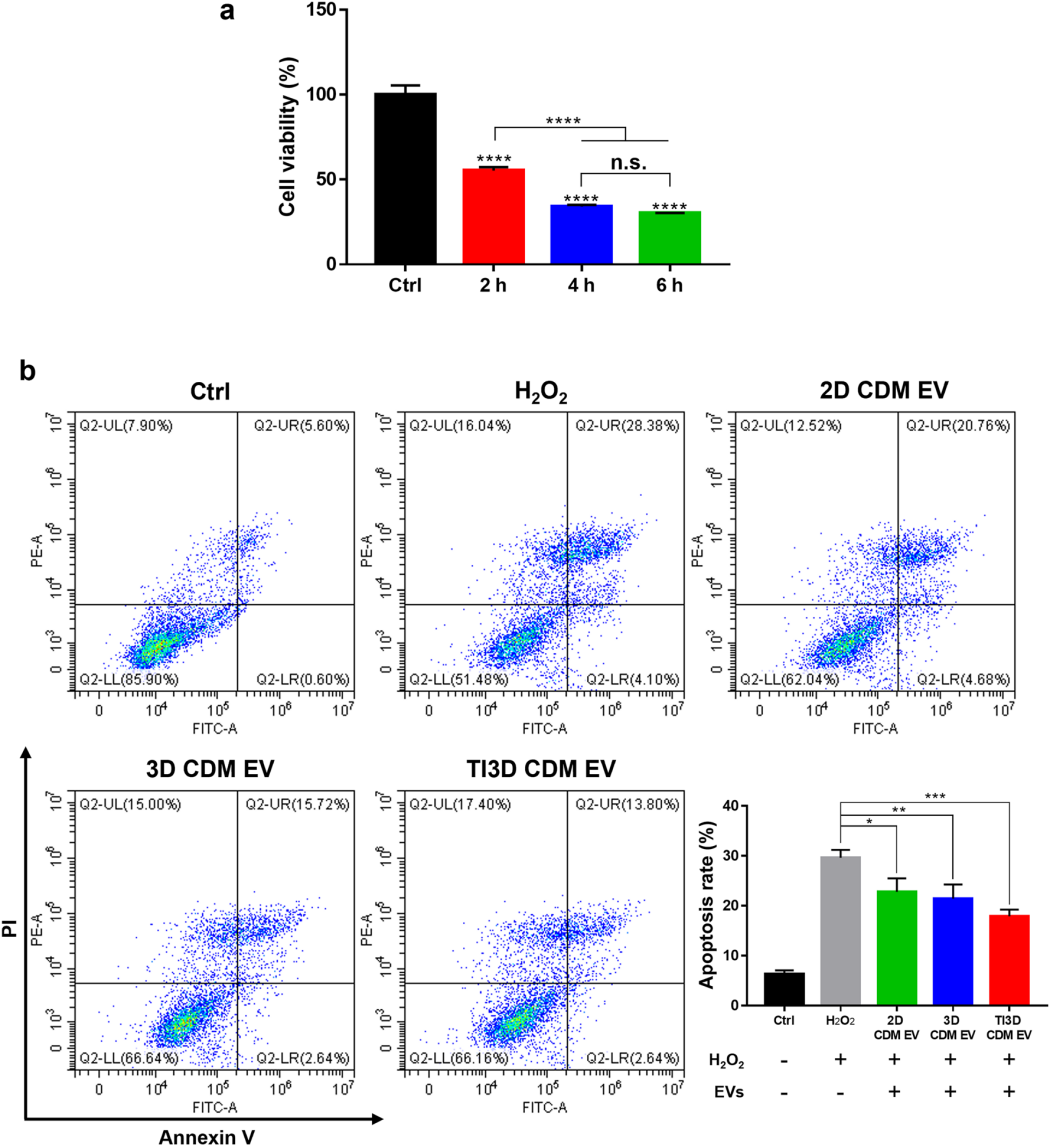
Anti-apoptotic effects of EVs.** a** Screening the optimum concentration of H_2_O_2_ for apoptotic cell modeling. **b** The plots of PI/Annexin V of H_2_O_2_ treated HK2 with EVs incubation and calculated the anti-apoptotic properties of EVs (values are presented as mean ± SD (n = 3) and statistical significance was obtained with one-way analysis of ANOVA with Tukey’s multiple comparison post-test (*p < 0.05; **p < 0.01; ***p < 0.001; ****p < 0.0001))

The oxidative stress characterized by excessive production of reactive oxygen species (ROS) is an important molecular mechanism causing apoptosis. It is usually referred to as the imbalance between ROS production and antioxidant defense, which finally induces cell dysfunction and tissue damage [66, 67]. The strategy to scavenge ROS is to prevent apoptosis-related diseases, such as atherosclerosis, diabetes mellitus, insulin resistance, and cancer [68]. Furthermore, as one of the most representative members of ROS, hydrogen peroxide (H_2_O_2_) results in oxidative damage and thereby leads to the functional loss of cells with aberrantly high levels [69]. Moreover, NF-κB is known to regulate the two kinds of proapoptotic Bcl2 families, Bax and Bcl-XS, to cause apoptosis [70]. Further, with the bioactivity of EVs on negative regulation of the apoptotic process as resulted in GO-BP analysis and NF-κB downregulation, the anti-apoptosis property of EVs was compared using flow cytometry analysis. The optimization of the incubation time of H_2_O_2_ for the apoptotic condition of HK2 cells was evaluated by time-dependent cell viability after treating the same amount of H_2_O_2_ (Fig. 7a). Additionally, anti-apoptotic rates of EVs were measured in HK2 cells with H_2_O_2_ treatments using flow cytometry (Fig. 7b). HK2 cells were exposed to H_2_O_2_ for 2 h to induce apoptosis. The administration of hUCMSC-derived EVs showed protective effects with a decrease in apoptosis rate in H_2_O_2_-treated apoptotic cells compared to the non-treated group (non-treated group: 28.38%, 2D CDM EV: 20.76%, 3D CDM EV: 15.72%, and TI3D CDM EV: 13.80%, respectively).

### Anti-fibrotic effects of EVs

**Fig. 8:**
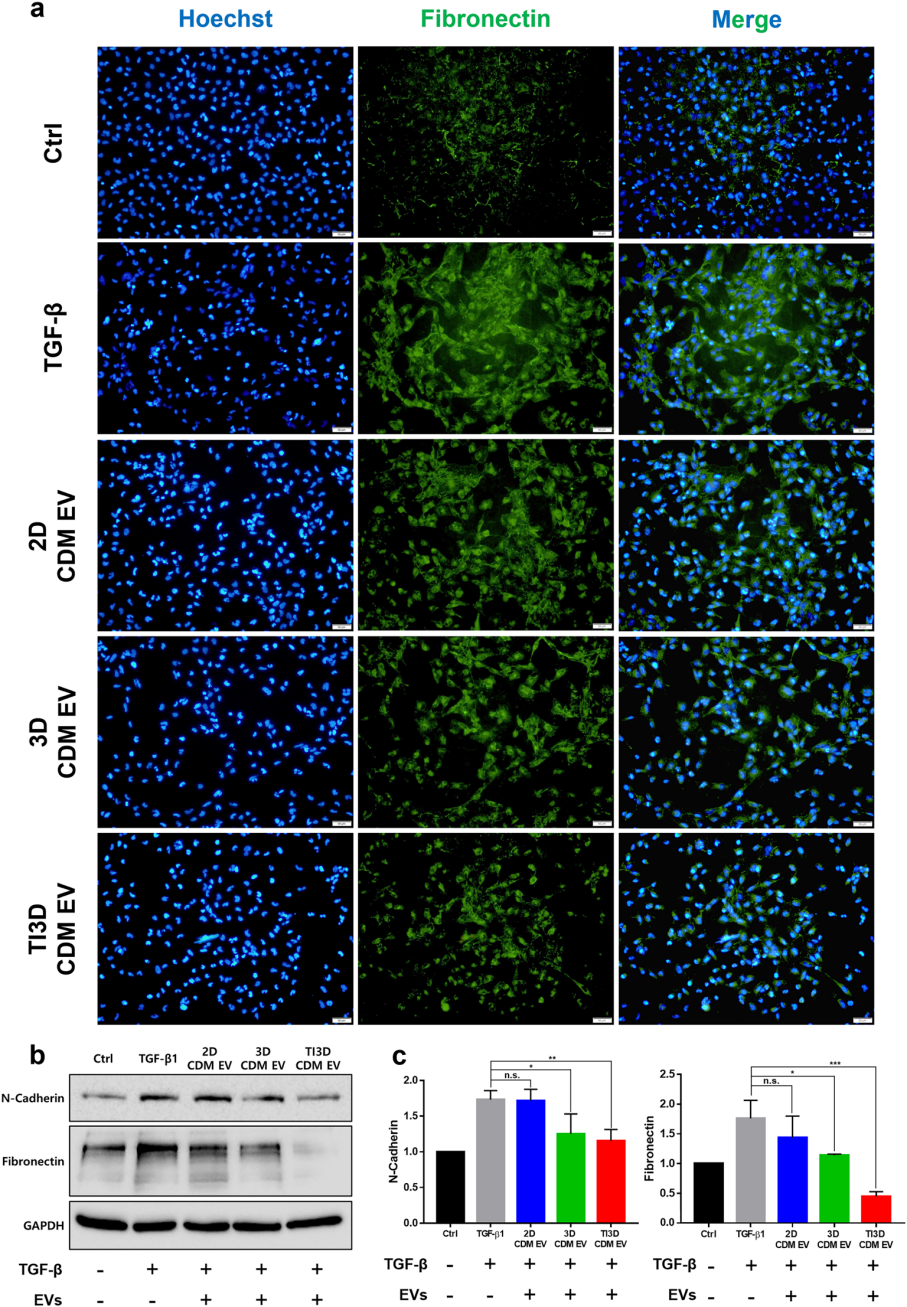
Anti-fibrotic properties of EVs.** a** The fluorescence-based immunocytochemistry of fibronectin expression of TGF-β induced fibrosis condition in HK2 cells. **b** Western blot analysis for representative fibrosis markers after EVs treatments in fibrosis induced-HK2 cells. **c** Quantification data for N-cadherin and fibronectin expression from Western blot analysis (analysis with Image J, values are presented as mean ± SD (n = 3) and statistical significance was obtained with one-way analysis of ANOVA with Tukey’s multiple comparison post-test (*p < 0.05; **p < 0.01; ***p < 0.001; ****p < 0.0001))

The inhibition of the epithelial-mesenchymal transition (EMT) of tubular epithelial cells enables the reduction of fibrosis, which is associated with the key mediator of inflammation, NK-κB activation [71–73]. Moreover, as the NF-κB suppression property for ameliorating fibrotic pathways, TI3D CDM EV was expected to have the most anti-fibrotic activity [74, 75]. The results of ICC visualized the intensity of representative fibrosis marker, fibronectin, in transforming growth factor *β* (TGF-*β*)-induced HK2 cell for fibrosis after EVs treatments. Figure 8a shows the expression level of fibronectin decreased with EVs treatments, especially with TI3D CDM EV administration. TI3D CDM EV-derived significant inhibition of TGF-*β* induced EMT markers, N-cadherin and fibronectin, was demonstrated using Western blot analysis (Fig. 8b). Furthermore, the intensity differences of N-cadherin and fibronectin were quantified by dividing with the intensity of GAPDH from Western blot analysis (Fig. 8c), which are correlated with the trends of ICC results.

## Conclusion

Many efforts on accelerating the production and bioactivities of EVs have been reported by modulating cell culture conditions in serum-containing and starvation conditions. Our previous reports suggested that the cell culture and EV isolations were optimized with high productivity, purity, and bioactivity using CDM compared to starvation condition, the gold standard condition for EV isolation without impurities from serum. Herein, we further modify the cell culture conditions and comparatively analyze the secretion and bioactivities of EVs in CDM. The secretion of EVs was elevated with culturing 3D systems (spheroids) and further promoted with TI priming of hUCMSCs. Further, while the total number of EVs was lower for 3D CDM than for 2D CDM due to the abundant proliferation rate of 2D cultured hUCMSC in CDM, the production yield of EVs per cell increased significantly. In addition, TI priming with maintaining cell viability also put forward the secretion and functionalities of EVs from 3D cultured hUCMSC in CDM. With increasing production yield, the expectations of improved regeneration-related bioactivities such as angiogenesis, wound healing, anti-inflammation, anti-apoptosis, and anti-fibrosis of EVs, resulted in bioinformatics analysis were demonstrated with cell-based assays. Moreover, by excluding side effects caused by impurities derived from serum components, it is expected that the value for utilities of MSC-derived EVs that can be applied clinically should be enhanced by tuning the culture conditions of MSC in CDM.

## Supplementary information

**Additional file 1. Fig. S1**. Estimation for thenumber of cells in 3D spheroids. **Fig. S2.** 3D spheroids formation.

## Data Availability

The datasets used and/or analysed during the current study are available from the corresponding author on reasonable request.
