# Supplementary material for: Bolstering the secretion and bioactivities of umbilical cord MSC-derived extracellular vesicles with 3D culture and priming in chemically defined media
Source: Nano Converg. 2022 Dec 19;9:57. doi: 10.1186/s40580-022-00349-z (PMC9761620; doi:10.1186/s40580-022-00349-z)
Supplement: Supplementary file 1 — Additional file 1. Fig. S1. Estimation for thenumber of cells in 3D spheroids. Fig. S2. 3D spheroids formation. [file 40580_2022_349_MOESM1_ESM.docx]

**Additional file 1**

**SI 1. Estimation for the number of cells in 3D spheroids.**

The average diameter of spheroids started at 124 μm with 5 × 10^5^ cells initially, and augmented to 135 μm after 4 days. Under the assumption that the spheroids have spherical structures, the number of cells could be calculated with regards to the amount of increasing volume compared to initial point.

**SI 2.**


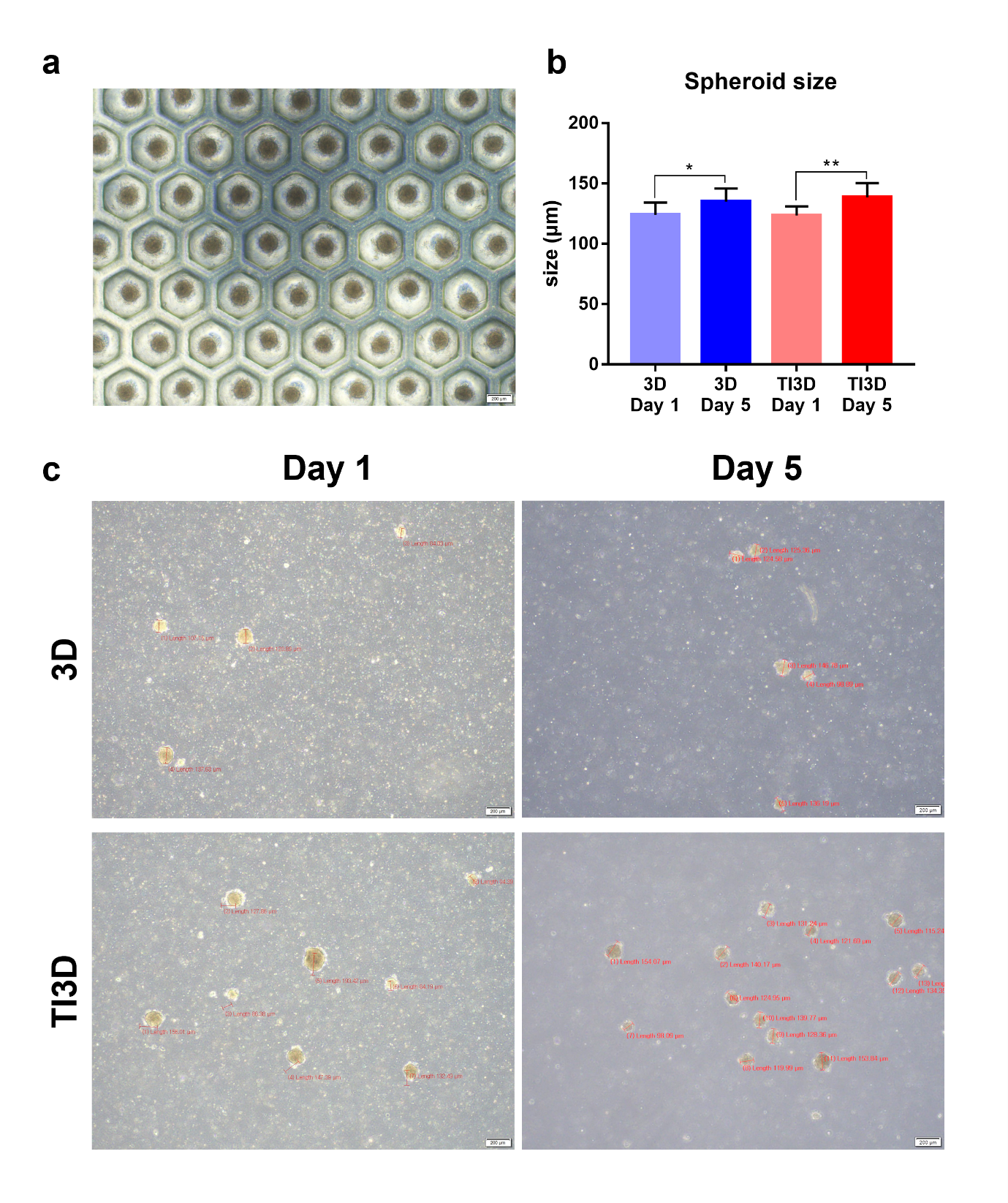


**SI 2. 3D spheroids formation**. **a** The bright field image of 3D spheroids cultured using StemFIT 3D^®^ plate. **b** Time-dependent size changes of spheroids with and without TI priming. **c** The representative images of 3D spheroids at 1 day and 5 days after spheroids formation. Scale bars equal to 200 μm. (Values are presented as mean ± SD (n = 3) and statistical significance was obtained with one-way analysis of ANOVA with Tukey’s multiple comparison post-test (**p* < 0.05; ***p* < 0.01; ****p* < 0.001; *****p* < 0.0001)).
